# *Terminalia ivorensis* demonstrates antioxidant properties and alters proliferation, genomic instability, and migration of human colon cancer cells *in vitro*

**DOI:** 10.1093/mutage/geae026

**Published:** 2024-10-23

**Authors:** Aliu Moomin, Rachel M Knott, Wendy R Russell, Mary P Moyer, Susan J Duthie

**Affiliations:** School of Pharmacy and Life Sciences, Robert Gordon University, Aberdeen, AB10 7GJ, United Kingdom; Rowett Institute, University of Aberdeen, Aberdeen, AB25 2ZD, United Kingdom; School of Pharmacy and Life Sciences, Robert Gordon University, Aberdeen, AB10 7GJ, United Kingdom; Rowett Institute, University of Aberdeen, Aberdeen, AB25 2ZD, United Kingdom; INCELL Corporation LLC, 12734 Cimarron Path, San Antonio, TX 78249, United States; School of Pharmacy and Life Sciences, Robert Gordon University, Aberdeen, AB10 7GJ, United Kingdom

**Keywords:** *Terminalia ivorensis*, antioxidant activity, cell growth, cell viability, DNA damage/repair, cell migration

## Abstract

Colorectal cancer is a global killer that causes approximately 940 thousand deaths annually. *Terminalia ivorensis* (TI) is a tropical tree, the bark of which is used in African traditional medicine for the treatment of diabetes, malaria, and ulcer. This study investigated TI as a potential anticancer agent in human colon cells *in vitro*. TI was extracted sequentially with petroleum ether, chloroform, ethyl acetate, and ethanol. Antioxidant activity was assessed by DPPH and FRAP, and differential effects on cell viability, growth, DNA damage, DNA repair, and migration were measured in human colon cancer cells (CaCo-2) and/or non-cancerous human colonocytes (NCM460). The TI phytochemicals most strongly associated with these effects were identified by partial least-squares discriminant analysis. DPPH and FRAP activity was highest in TI ethyl acetate and ethanol extracts (*P* = .001). All TI extracts significantly inhibited cell viability and growth and induced DNA damage and inhibited DNA repair in both cell models. The majority of TI extracts were significantly (*P* = .01) more toxic to cancer cells than non-cancerous colonocytes. DNA repair was significantly (*P* = .001) inhibited in CaCo-2 cells by ethyl acetate extract compared with NCM460 cells. Migration was also significantly inhibited (*P* < .001) in CaCo-2 by ethyl acetate (80%) and ethanol extracts (75%). Specific benzoic acids, flavonoids, and phenols were identified to be strongly associated with these effects. TI displayed strong antioxidant activity and specific anticancer effects by inducing cell death and DNA damage, and by inhibiting DNA repair, cell proliferation, and migration.

## Introduction

Colorectal cancer (CRC) is the third-most prevalent, and second-most deadly cancer worldwide, with 2.2 million new cases and 940 thousand deaths recorded in 2020 [1]. Early diagnosis and medical treatments, such as chemotherapy, radiotherapy, and surgery, are used to reduce cancer mortality [2]. Many chemotherapy drugs such as 5-fluorouracil, anthracycline, and methotrexate are used in the treatment of numerous cancers including breast, colon, leukaemia, lung, and stomach cancers, but none are entirely effective or safe [3,4]. Toxicity is the major problem associated with established cancer therapy, with chemo- and radiotherapy strongly linked with serious side effects, such as a high risk of bleeding, blood clots, headache, hypertension, skin inflammation, gut perforations, and secondary cancers [3,5]. Cancer cells can also develop resistance to therapeutic drugs [5]. While there have been significant advances in the treatment of cancer, there remains an urgent need to identify and validate safer and effective alternatives [3].

Natural products (from plants, animals, and microorganisms) have been studied extensively as potentially safe and cost-effective treatments for cancer. Some of these natural products exhibit anticancer properties by interfering with the initiation, development, and progression of cancer, and by modulating key carcinogenic mechanisms including inducing apoptosis, inhibiting continuous cellular proliferation, angiogenesis, and metastasis [6]. A tropical tree, *Terminalia ivorensis* (TI), is used in traditional medicine for the treatment of diuresis, malaria, and ulcers [7–9]. Several *in vitro* studies have also observed antibacterial, antioxidant, antifungal, and anti-plasmodial properties of TI [10–12], while in mice and rat models, TI has anti-inflammatory, anti-nociceptive, nephro-, and hepatoprotective effects [13–15]. We have recently performed a comprehensive identification and analysis of the phytochemicals contained with several sequential solvent extracts from TI and shown that different solvent extracts of TI produce different phytochemical profiles [7]. In traditional African medicine, herbal products are usually extracted with different solvents for different medicinal purposes. For instance, ethanol extract of TI shows anti-plasmodial and anti-nociceptive effects while hydroalcoholic extract shows anti-fungal effects [10–12]. Thus, in this study, the ability of different solvent extracts from TI to influence several key hallmarks of the carcinogenic process was determined in both cancerous and non-cancerous colon cell models.

NCM460 cells are human colon epithelial cells that were isolated originally from the normal colonic mucosa of a 68-year-old Hispanic male [16]. While primary colon mucosal epithelial cells have a lifespan of only a few days, NCM460 cells, due to the presence of a mutated TP53 gene, have become immortalized [16,17]. NCM460 cells are not malignantly transformed, and retain many normal mucosal colonocyte characteristics, including expression of the epithelial cell antigens cytokeratin and villin [16]. The NCM460 cell line has been used extensively to investigate the absorption and metabolism of folate and other B vitamins and to investigate how protective phytochemical compounds in the diet act differentially against normal and cancerous colon cells *in vitro* [17]. Therefore, NCM460 cells are a good model for normal colonic epithelium.

CaCo-2 cells, originally isolated from a colon carcinoma of a 72-year-old Caucasian, are an established model for human colon cancer and have been used to study the effects of the microbiota or their metabolites on the barrier function of the intestinal epithelium; to investigate pathways involved in the transport of drugs or food components across the intestinal epithelium and to study the potential toxicity of drugs or food metabolites in the intestinal mucosa [18].

This study investigated the *in vitro* antioxidant potential of TI and the differential effect of TI extracts (from different solvents) on CaCo-2 and NCM460 cell viability, growth, DNA damage, DNA repair and cell migration.

## Material and methods

### Preparation of TI extracts

Bark samples of TI were collected from Asakraka Kwahu in the Eastern region of Ghana in February 2018. The samples were washed thoroughly with tap water and air dried at room temperature for 2 weeks. The dried samples were broken down into smaller pieces with a domestic food processor and then powdered using a freezer mill (SPEX sample prep 6870, Fisher Scientific, Loughborough, UK). The powdered samples were stored at −80°C until required for isolation of phytochemicals. TI sample extraction and identification of phytochemicals was as described previously [7,19]. The extracts were evaporated to dryness and reconstituted in dimethyl sulfoxide (DMSO) for testing in cells.

### Antioxidant activity of TI extracts

#### DPPH Scavenging activity

The 1,1-diphenyl-2-picrylhydrazyl (DPPH) assay was used to determine the antioxidant scavenging activity of all TI extracts [20]. Chloroform and petroleum ether extracts were used at a final concentration of 1.3–333.3 µg/ml and ethanol and ethyl acetate extracts at 0.26–66.7 µg/ml.

#### Ferric reducing antioxidant power (FRAP)

The FRAP assay was performed as described previously [21]. Serial dilutions of Trolox (standard) were prepared in water:ethanol (4:10) with concentrations ranging from 31.2 to 312.5 µg/ml. Each TI extract was also prepared in water:ethanol (4:10) with concentrations ranging from 31.2 to 1000 µg/ml. The results are expressed as Trolox equivalents as TE/µg dry sample [21].

### Maintenance and routine culture of human colon cells *in vitro*

CaCo-2 cells were purchased from the European Collection of Authenticated Cell Cultures (ECACC No. 86010202, lot 17H003, passage 8-12) and grown in monolayer or multilayer culture in low glucose (1 g/L) DMEM supplemented with 20 % (v/v) FBS, 1% (v/v) non-essential amino acids (NEAAs), and 1% (v/v) of a solution of penicillin (100 U/ml): streptomycin (100 µg/ml) [22].

NCM460 cells were obtained from INCELL (San Antonio, TX, passage 3-10) under a Material Transfer Agreement (Proprietary Cell Lines | INCELL). In order to allow for a direct comparison of the influence of TI on biomarkers in an established *in vitro* model of colon cancer and in non-malignantly transformed human colonocytes, NCM460 cells were grown as a monolayer culture in high glucose (4.5 g/L) DMEM supplemented with 10% (v/v) FBS, 1% (v/v) NEAAs, and 1% (v/v) penicillin (100 U/ml): streptomycin (100 µg/ml) (Culture Media | INCELL) [17].

### The effect of TI on colon cell viability and growth *in vitro*

#### Cell viability

The impact of TI on cell viability was measured in both CaCo-2 and NCM460 cells using the MTT assay [23]. Cells were seeded at 7.5 × 10^3^ cells/well in 96-well microtiter tissue culture plates in complete culture medium and incubated at 37°C for 24 h in 95% air /5% CO_2_. After 24 h, TI extracts (0–5.0 mg/ml final concentration) were added and the cells incubated at 37°C for a further 24 h. The medium was then removed and methyl thiazol tetrazolium bromide (MTT, Fisher Scientific, Loughborough, UK) solution (1 mg/ml) was added and the plate was incubated at 37°C for 4 h. The MTT was removed, DMSO (100%) was added for 20 min, and the absorbance was read at 560 nm with a spectrophotometer.

#### Cell growth

CaCo-2 or NCM460 cells were seeded at 7.5 × 10^4^ cells/well in 12-well microtiter tissue culture plates and incubated at 37°C for 24 h in 95% air/5% CO_2_ [22]. After 24 h, TI extracts (0–5.0 mg/ml final concentration) were added and the cells were incubated at 37°C for another 24 h. The medium was subsequently removed, and the cells were washed with sterile PBS and incubated with 0.25 % trypsin (v/v) 0.03% EDTA at 37°C for 5 min. Complete medium (0.5 ml) was used to stop the trypsin digestion and the cells were counted with a haemocytometer [22].

### The influence of TI on genomic stability on colon cells *in vitro*

#### DNA single-strand breakage

The effect of TI on DNA single-strand breakage (SSB)/DNA integrity was measured using single-cell gel electrophoresis (SCGE; comet assay) [22]. CaCo-2 or NCM460 cells were grown on 24-well plates at a density of 7 × 10^5^ cells/well and incubated at 37°C for 24 h. Cells were incubated with TI chloroform, ethanol, ethyl acetate, or petroleum ether extract at a final concentration of 0–5.0 mg/ml at 37°C for 24 h in 5% CO_2_/95% air, harvested with trypsin-EDTA, and counted using a haemocytometer. Cells were then pipetted into PBS (1 ml final volume) and centrifuged at 200 g for 5 min at 4°C. The supernatant was discarded, and the pellet resuspended in 85 µl of 1% (w/v) low melting point agarose and pipetted onto a frosted microscope slide precoated with 1% (w/v) high melting point agarose. The gels were allowed to set at 4°C for 10 min, and the slides incubated in lysis solution [2.5 M NaCl, 0.1 M EDTA, 10 mM Tris, NaOH to pH 10.0, and 1% (v/v) Triton X-100] at 4°C to remove cellular protein and lipids (membranes). The slides were incubated in lysis solution in separate boxes to prevent the potential leaching of higher concentrations of TI into lower concentrations. Slides were then aligned in a 260 mm wide horizontal electrophoresis tank containing electrophoresis solution (1 mM EDTA and 300 mM NaOH, pH 12.7) for 40 min before electrophoresis at 4°C for 40 min at 25 V and 999 mA. Slides were then washed 3 × 5 min each at 4°C in neutralizing buffer (0.4 M Tris-HCl, pH 7.5) and stained with 20 µl of 4,6-diamidine-2-phenylindol dihydrochloride (DAPI; 1 mg/ml stock solution). Comet images were analysed visually, and the resulting scores represented the level of SSBs. This was carried out according to a well-established scoring system where 100 images per gel (with each treatment at least in duplicate) were assigned a value of 0, 1, 2, 3, or 4 (from undamaged to maximally damaged) depending on the intensity of the fluorescence in the comet tail. Thus, the total score for 100 comets ranged from 0 to 400 from all undamaged cells to maximally damaged cells, respectively [22].

#### DNA strand break repair (SBR)

The effect of TI on DNA SBR of oxidant-induced DNA damage was as described previously [22]. CaCo-2 or NCM460 cells were incubated in 24-well plates at a density of 7.4 × 10^5^ cells/well at 37°C for 24 h. Cells were then exposed to TI extract (final concentration of 0.25 mg/ml in medium) at 37°C for a further 24 h. DMSO (0.5 %) was used as control. The cells were then washed twice with PBS before exposure to H_2_O_2_ (30 µM) for 5 min on ice. Residual H_2_O_2_ was removed by washing the cells twice with PBS. The cells were then incubated in serum-free medium at 37°C in a humidified atmosphere of 5% CO_2_ / 95 % air before harvesting with trypsin-EDTA after 0, 30 or 60 min, and DNA SBR capacity analysed by SCGE. The DNA SBR was measured in terms of DNA recovery after H_2_O_2_-induced damage [22].

### Effect of TI on Caco-2 cell migration.

**Figure 1.**
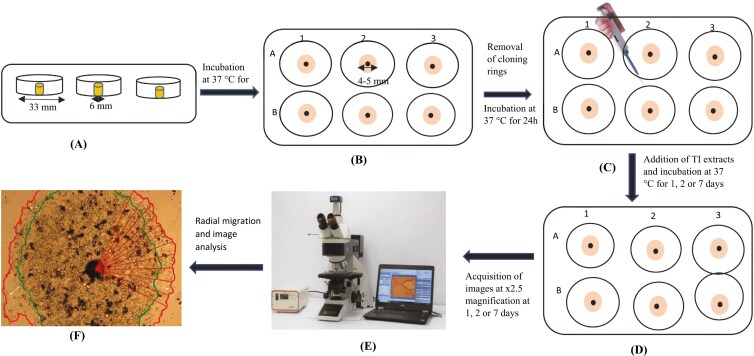
Formation of circular monolayers of CaCo-2 cells and the acquisition of cellular images for migration. (A) side view of cloning rings placed centrally in wells, 4 × 105 cells/ring pipetted into rings and incubated at 37°C for 4–6 h, (B) Aerial view of cells after removal of cloning rings and addition of 2 ml of medium for a further incubation at 37°C for 24 h, (C) Addition of dimethyl sulfoxide (DMSO), or hydroxyurea (HU) or TI extract and HU, acquisition of images at day 0 of experiment and a further incubation at 37°C for 1, 2, or 7 days and (D and E) Acquisition of cellular images at ×2.5 magnification after 1, 2, or 7 days of treatment with DMSO and HU or TI extract and HU [image (E) adapted from bostonind.com], (F) Radial migration and image analysis after treatment of CaCo-2 cells with DMSO and HU or TI extract and HU. The inner circular outline is the image before treatment (day 0), and the outer circular outline is the image after treatment for 1, 2 or 7 days. The arrows show the direction of cell migration.

The effect of TI on CaCo-2 cell migration was as described earlier [24]. Cells were seeded in complete medium in a 6-well plate within sterile cloning rings (6 × 8 mm, Fisher Scientific, UK) at a density of 4 × 10^5^ cells/ring and incubated at 37°C for 4–6 h. The cloning rings were removed, and fresh complete medium was added to the cells. Images of the cells were acquired (Time 0) to note cell attachment and the formation of monolayers (Fig. 1a and b). The cells were then incubated at 37°C for a further 24 h TI (petroleum ether, chloroform, ethyl acetate or ethanol extracts; 0.25 mg/ml) and hydroxyurea (HU; 5 mM) were added concurrently to the cells. DMSO (0.5%) was used as solvent control and HU was used to stop cell growth but still get migration of the cells (Fig. 1c and d). Digital images of cells were acquired using a Leica DMI 4000B microscope (Leitz Wetzlar, Germany) at a magnification of ×2.5 to observe cells for attachment and formation of monolayers after the addition of TI extracts at Time 0, 1, 2, or 7 days of treatment (Fig. 1e).

#### Image analysis

Photomicrographs for cell migration were analysed using an image processing and analysis software (ImageJ software version 1.51r). Briefly, a 90° angle was generated on the image acquired at day 0, and 10 radii were drawn from a central reference mark. The procedure was repeated for the same well after incubation for 1, 2, or 7 days (Fig. 1f). Net migration was calculated using the difference between the average radii at 1, 2, or 7 days (Fig. 1f), and the average radii at day 0 and the data presented as mean ± SEM [25].

Net migration = r2—r1, where r1 is the average of radii of circular image (green outline) at day 0 and r2 is the average of radii of image (red outline) after treatment with control or extract for 1, 2, or 7 days (Fig. 1f).

### Data analysis

Effects of TI extracts on antioxidant activity, cell viability, proliferation, genomic stability, and cell migration were compared using one-way analysis of variance (ANOVA) followed by Bonferroni multiple comparison test. A difference or effect was considered significant if *P* < .05. The IC_50_ and LC_50_ of the TI extracts were calculated for cell viability, DNA SSB and rate of DNA SBR to determine the fold change in cyto- and genotoxicity on NCM460 and CaCo-2 cell lines.

Partial least squares—discriminant analysis (PLS-DA) was used to determine the relationship between the major identified phytochemicals present within each TI extract, and DNA SBR rate, and CaCo-2 cell migration. Based on the measured effects, a plot was constructed for TI extract at a concentration of 0.25 mg/ml to represent those phytochemicals with the strongest impact on DNA SBR and CaCo-2 cell migration. A 50-cm diameter circle was drawn at the negative side of the graph showing the strongest measured inhibitory effect. Phytochemicals located within each circle were selected as those with the greatest impact on DNA SBR or CaCo-2 cell migration.

## Results

### Antioxidant activity of TI extracts

The concentration of TI at which 50% of DPPH radicals was scavenged (IC_50_) was calculated as a measure of antioxidant activity. The DPPH IC_50_ for TI ethanol extract (TIEE) and ethyl acetate extract (TIEaE) were significantly lower (*P* = .0001) compared to the IC_50_ for petroleum ether extract (TIPE) or chloroform extract (TICE). Similarly, TIEE and TIEaE showed significantly (*P* = .001) higher ferric-reducing antioxidant power (FRAP) compared to TIPE or TICE (Table 1).

**Table 1.** Comparison of antioxidant activity, IC_50_ (mg/ml) for TI extract on viability and growth, and LC_50_ (mg/ml) for DNA SSB in CaCo-2 and NCM460 cells.

	Antioxidant activity		IC_50_ (mg/ml) for cell viability		IC_50_ (mg/ml) for cell growth		LC_50_ (mg/ml) for DNA SSB	
TI	DPPH (IC50, µg/ml)	FRAP (IC_50_, TE/µg of TI dry sample)	NCM460	CaCo-2	NCM460	CaCo-2	NCM460	CaCo-2
Petroleum ether extract (TIPE)	1163.3 ± 6.2	3.8 ± 0.5	2.54	1.96	2.53	1.94	1.51	0.58
Chloroform extract (TICE)	1617.0 ± 186.3	2.1 ± 0.5	2.21	1.69	2.82	3.65	0.40	1.27
Ethyl acetate extract (TIEaE)	9.4 ± 0.1***	156.3 ± 0.9***	7.66	3.05	2.63	2.97	0.81	0.70
Ethanol extract (TIEE)	7.9 ± 0.8***	119 ± 0.7***	6.74	3.99	2.60	2.38	0.39	0.26

Data are presented as mean ± SEM, *n* = 3, ***P < .001 as compared to the TIPE or TICE by one-way ANOVA followed by Bonferroni’s multiple comparison test. TE is Trolox equivalents.

### The effect of TI on colon cell viability and growth *in vitro*

#### Cell viability

**Figure 2.**
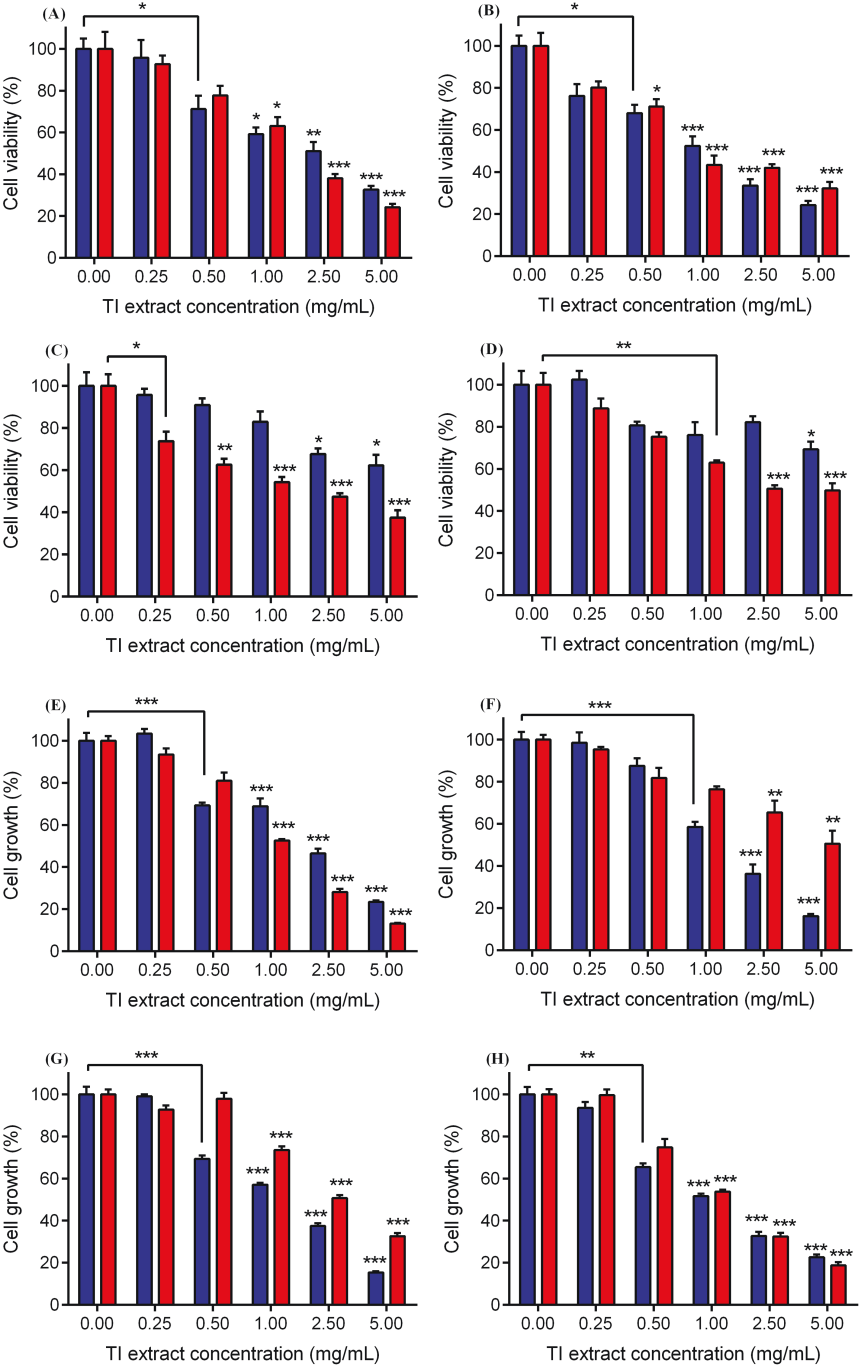
(a-d) Effect of TI extracts on CaCo-2 or NCM460 cell viability after 24 h treatment with (A) TI petroleum ether extract—TIPE, (B) TI chloroform extract—TICE, (C) TI ethyl acetate extract—TIEaE and (D) TI ethanol extract—TIEE and (e-h) shows the effect of TI extracts on CaCo-2 or NCM460 cell growth after 24 h treatment with (E) TIPE, (F) TICE, (G) TIEaE and (H) TIEE. Data are presented as mean ± SEM; *n* = 3; *P < 0.05; **P < 0.01 and ***P < 0.001 as compared to the appropriate DMSO control by one-way ANOVA followed by Bonferroni’s multiple comparison test.

As the concentration of all four TI extracts increased above 0.5 mg/ml, there was a corresponding decrease in the viability of both CaCo-2 and NCM460 cells. TICE showed the greatest effect on viability in both cancer and non-cancerous human colon cells followed by TIPE, TIEaE, and TIEE (Fig. 2a–d). CaCo-2 cells were more susceptible to TI extract (TICE and TIEE) at lower concentrations (IC_50_) while NCM460 cells were unaffected. Treatment with TICE or TIEaE at 0.5 mg/ml significantly (*P* < .05) decreased viability only in CaCo-2 cells, while TIEE at the same concentration significantly (*P* = .01) decreased viability only in NCM460 cells (Fig. 2b and c). Treatment with TIEE or TIEaE (IC_50_) was approximately twice as toxic in CaCo-2 cell viability as compared to NCM460 cells. The IC_50_s for TIEaE (7.66 mg/ml) and TIEE (6.74 mg/ml) were also approximately two times higher in NMC460 cell viability than for CaCo-2 cells (TIEaE = 3.05 mg/ml and TIEE = 3.99 mg/ml) (Table 1).

#### Cell growth

Treatment with TIPE (0.5 mg/ml) significantly (*P* < .05) inhibited proliferation in both NCM460 and CaCo-2 cells, while TICE and TIEaE at the same concentration significantly (*P* < .05) decreased growth only in NCM460 cells and TIEE at 1 mg/ml significantly (*P* < .01) decreased growth only in CaCo-2 cells. All TI extracts significantly (*P* < .05) inhibited growth in both cell models at concentrations ≥ 2.5 mg/ml (Fig. 2e–h).

A higher concentration (IC_50_ = 3.65 mg/ml) of TICE was required to reduce the growth of CaCo-2 cells by 50% (IC_50_), whereas a lower concentration (IC_50_ = 1.94 mg/ml) of TIPE was required to reduce the growth of CaCo-2 cells by 50%. CaCo-2 cells were more susceptible to TIPE at lower concentrations while NCM460 cells were unaffected at these concentrations (Table 1).

### The influence of TI on genomic stability of colon cells *in vitro*

#### DNA single-strand breakage

**Figure 3.**
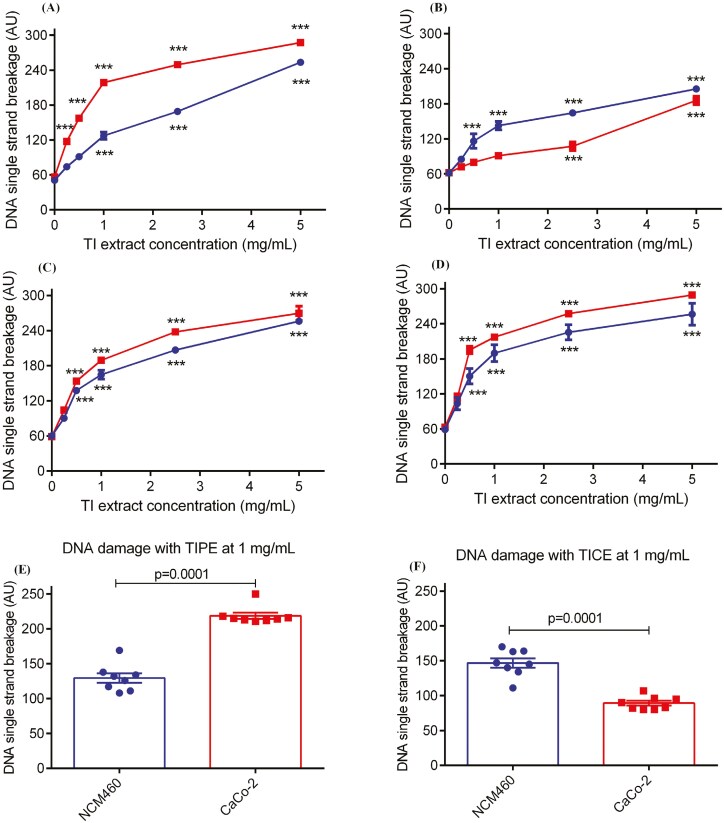
Effect of TI extract on CaCo-2 or NCM460 DNA strand breakage after 24 h treatment with (A) TI petroleum ether extract—TIPE, (B) TI chloroform extract—TICE, (C) TI ethyl acetate extract—TIEaE and (D) TI ethanol extract—TIEE, and (E and F) shows comparison of DNA damage in NCM460 versus CaCo-2 cells after exposure to TIPE and TICE at 1 mg/ml. Data are presented as mean ± SEM, *n* = 8; ***P < 0.001 as compared to DMSO control cells by one-way ANOVA followed by Bonferroni’s multiple comparison test.

Treatment with TIPE (0.25 mg/ml) significantly (*P* = .001) induced DNA SSBs only in CaCo-2 cells (Fig. 3a). SSB was increased non-specifically in both cell models at concentrations of all TI extracts above 2.5 mg/ml (*P* = .001) (Fig. 3a–d). DNA SSB was significantly (*P* < .01) higher in CaCo-2 cells than in NCM460 cells when the cells were exposed to TIPE at concentrations of 0.5–2.5 mg/ml (Fig. 3a and e). Conversely, significantly (*P* = .0001) higher DNA damage was measured in NCM460 when compared with CaCo-2 cells after treatment with TICE at concentrations of 0.5–2.5 mg/ml (Fig. 3b and f). TIEE induced the highest genotoxicity in both CaCo-2 (LC_50_ = 0.26 mg/ml) and NCM460 cells (LC_50_ = 0.39 mg/ml) (Table 1). TIPE caused approximately two times higher genotoxicity in CaCo-2 (LC_50_ = 0.58 mg/ml) compared to NCM460 (LC_50_ = 1.51 mg/ml) while TICE caused three times lower toxicity in CaCo-2 (LC_50_ = 1.27 mg/ml) than NCM460 (LC_50_ = 0.40 mg/ml) cells (Table 1).

#### DNA strand break repair (SBR)

**Figure 4.**
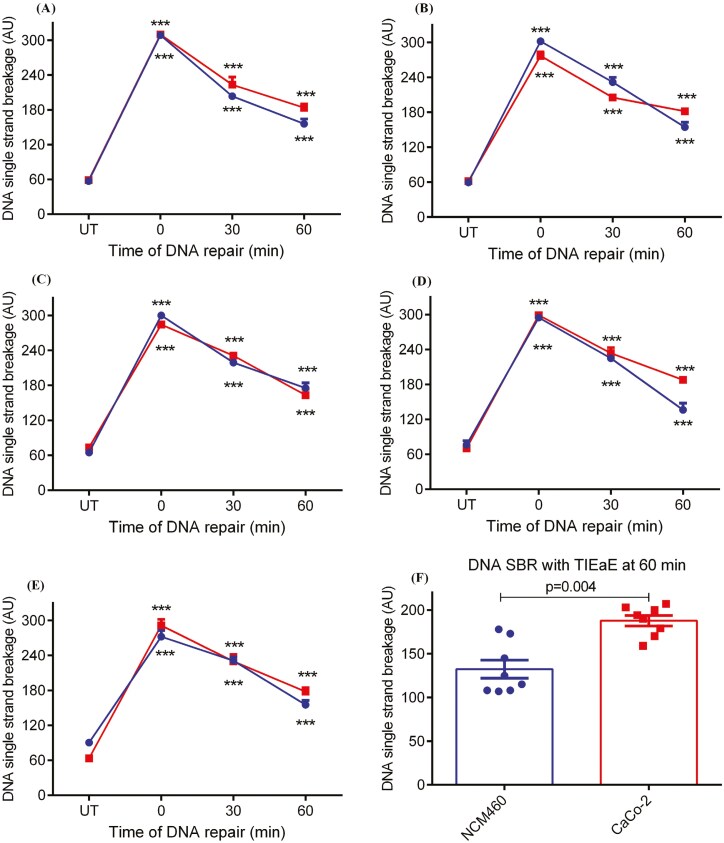
Effect of TI extract on CaCo-2 or NCM460 DNA strand break repair (SBR) after 24 h treatment with (A) solvent control (dimethyl sulfoxide, DMSO), (B) TI petroleum ether extract—TIPE, (C) TI chloroform extract—TICE, (D) TI ethyl acetate extract—TIEaE and (E) TI ethanol extract—TIEE, and (F) shows comparison of DNA SBR in NCM460 versus CaCo-2 cells after exposure to TIEaE and TICE at 0.25 mg/ml. Data are presented as mean ± SEM; *n* = 8; ***P < 0.001 as compared to untreated (UT) control (no H2O2 and TI extract) by one-way ANOVA followed by Bonferroni’s multiple comparison test.

As expected, there was a significant (*P* < .001) increase in DNA SSB in both colon cell models after exposure to H_2_O_2_. This was followed by a repair of DNA SSB after 30 and 60 min (Fig. 4a—e). The SBR was higher in NCM460 cells compared with CaCo-2 cells, as indicated by the lower level of SSB remaining at 60 min (Fig. 4f).

TI extract affected DNA SBR differently in the two colonocyte cell lines. DNA SBR activity was significantly (*P* < .01) higher in NCM460 cells exposed to TIEaE compared to the DMSO control-treated cells. This increase in DNA SBR in response to TIEaE was not observed in CaCo-2 (Fig. 3h). Conversely, the rate of SBR in TIEE-treated NCM460 cells was approximately three times lower after 30 min (1.37 AU/min) and twice as lower after 60 min (1.95 AU/min) when compared with DMSO control (3.51 and 2.55 AU/min respectively). DNA SBR in TICE-treated CaCo-2 cells was approximately twice as low after 30 min (1.78 AU/min) when compared with DMSO control (2.89 AU/min) (Table 2).

**Table 2.** Effect of TI extract on the DNA stand break repair rate (AU/min) in NCM460 and CaCo-2 cells measured at 30 and 60 min after removal of H_2_O_2_ from the cells.

	NCM460 cells (AU/min)		CaCo-2 cells (AU/min)	
Treatment/Time	30 min	60 min	30 min	60 min
Control	3.51	2.55	2.89	2.10
TIPE	2.34	2.46	2.38	1.59
TICE	2.70	2.08	1.78	2.02
TIEaE	2.33	2.64	2.18	1.85
TIEE	1.37	1.95	2.05	1.89

#### Effect of TI phytochemical metabolites on DNA SBR in colon cells

**Figure 5.**
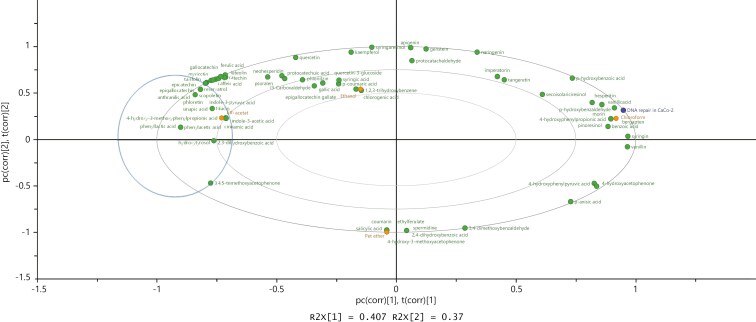
Association between TI extracts and DNA strand break repair (SBR) in CaCo-2 cells after 24-h treatments with TI extracts. The plot was colour coded as: deep blue for DNA repair; green for phytochemical compounds; and orange for TI extracts.

Based on the different effects of the TI extracts on DNA SBR in cancer versus non-cancer colon cell model, where treatment only with TIEaE inhibited DNA SBR only in CaCo-2 cells compared with NCM460 cells (Fig. 4d and f), the potential impact of specific phytochemicals present in TIEaE on DNA SBR was analysed by PLS-DA. The differential inhibition of SBR capacity by TIEaE in colon cancer-derived CaCo-2 cells, but not in NCM460 cells, was associated with the presence of 4-hydroxy-3-methoxyphenylpropionic acid; hydroxytyrosol and indole-3-acetic acid, which were not present in TICE, TIEE, or TIPE (Figure 5) [7].

### Effect of TI on CaCo-2 cell migration *in vitro*

**Figure 6.**
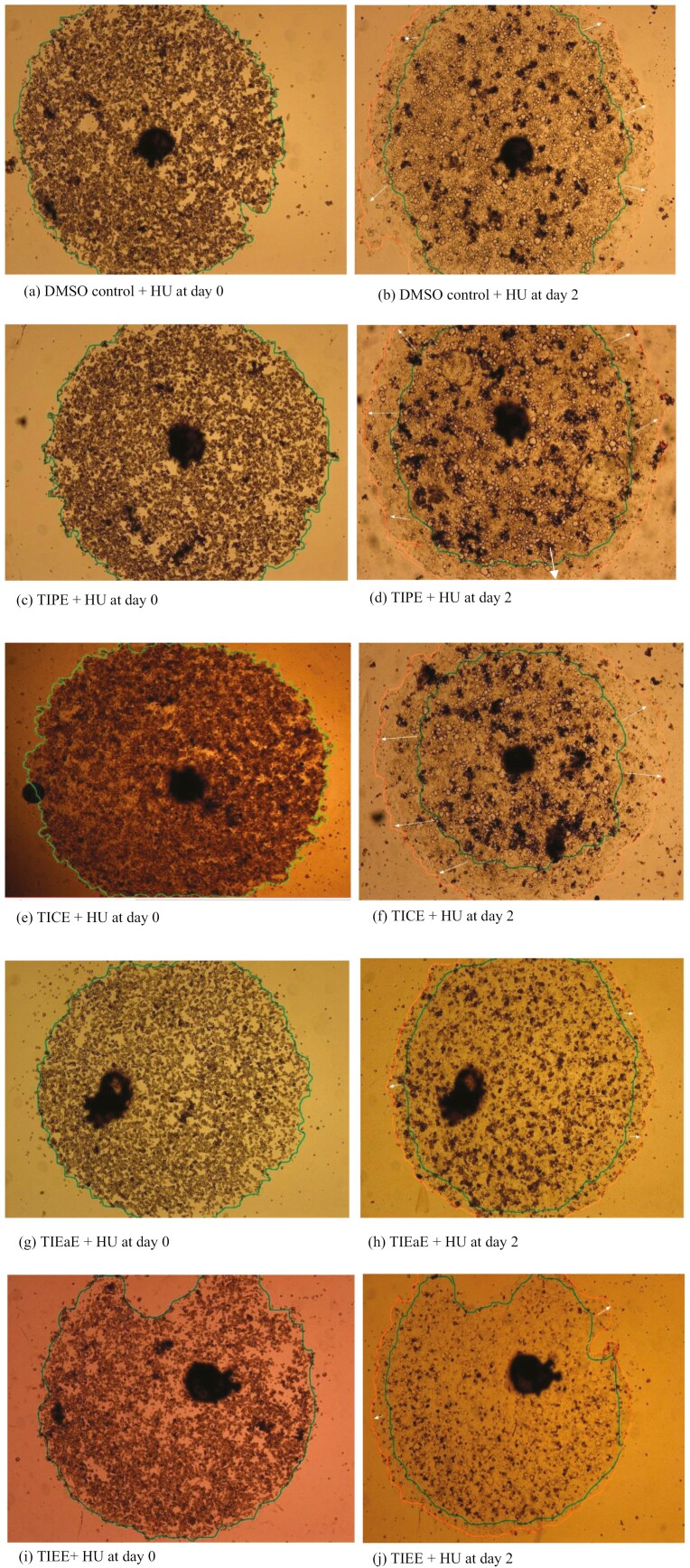
Circular monolayers of CaCo-2 cells treated with TI extract and hydroxyurea (HU). Inner circular outlines showing cells at day 0, outer circular outlines showing the leading edges of cell migration and white arrows showing the leading edges of migration at day 2 of treatment.

CaCo-2 cells were either treated with DMSO + HU (Control) or with TI + HU and cell migration was measured at 1, 2, and 7 days (representative images shown in Fig. 6). After 2 days, CaCo-2 cells had attached and started to migrate. The rate of migration in cells pre-treated with TIPE or TICE was similar to control cells (Fig. 6a–f). Conversely, a substantially reduced migration rate was observed in cells pre-treated with either TIEE or TIEaE (Fig. 6g–j).

**Figure 7.**
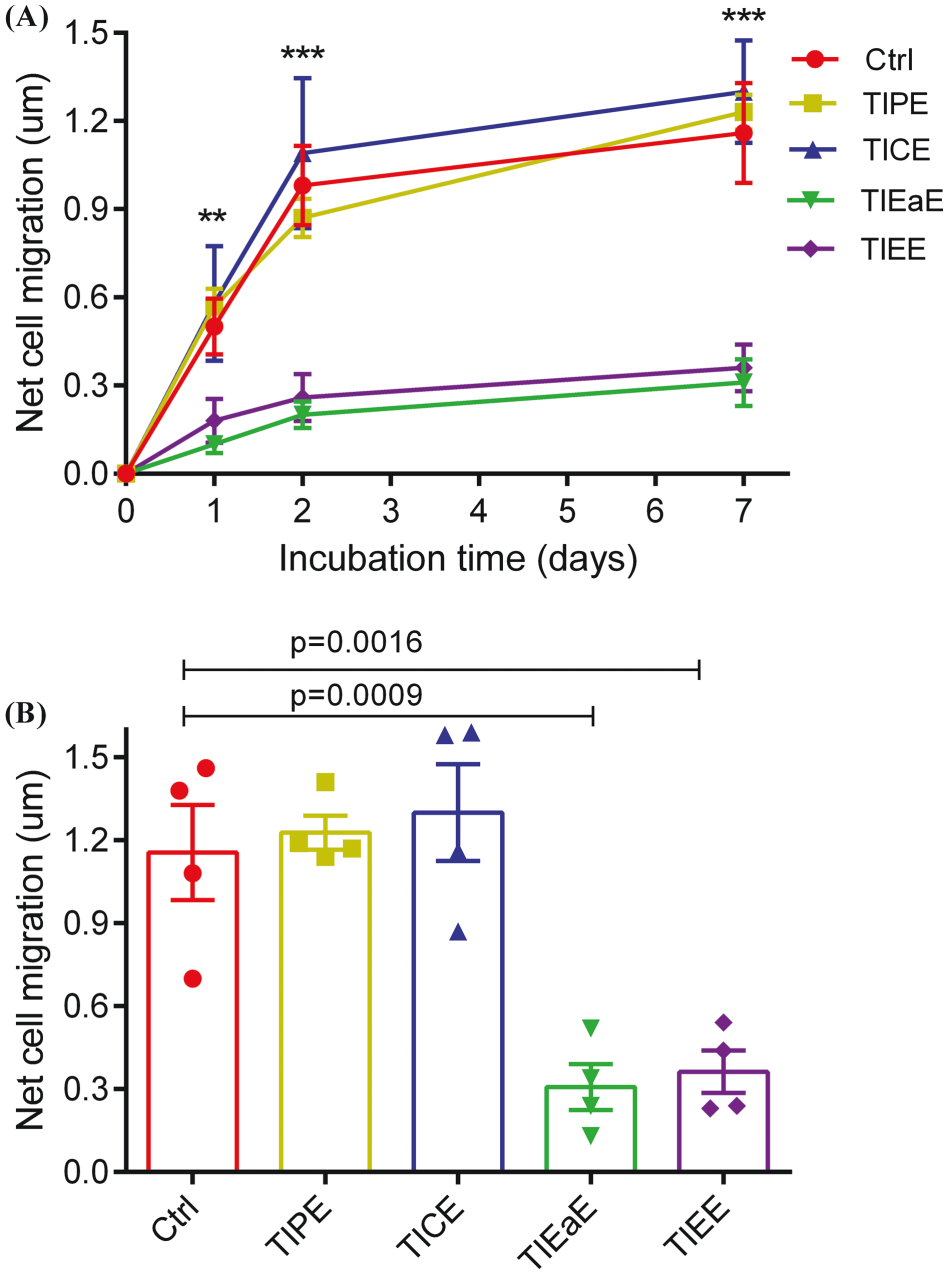
(A) Effect of TI extracts on CaCo-2 cell migration for all four TI extracts and control and (B) Comparison of CaCo-2 cell migration after treatment with TI extracts for 7 days. Dimethyl sulfoxide (DMSO) solvent control—Ctrl, TI petroleum ether extract—TIPE, TI chloroform extract—TICE, TI ethyl acetate extract—TIEaE and TI ethanol extract—TIEE, Data are presented as mean ± SEM; *n* = 4; **P < 0.01 and ***P < 0.001 as compared to time 0 by One-way ANOVA followed by Bonferroni’s multiple comparison test.

There was a significant (*P* < .001) time-dependent increase in migration in control cells (DMSO + HU), across the 7 days period with a similar effect measured in cells pre-treated with TIPE and TICE (Fig. 7b). On the other hand, pre-treatment with TIEE (*P* = .0016) or TIEaE (*P* = .0009) significantly and substantially inhibited Caco-2 cell migration at 7 days by approximately 80% and 75%, respectively (Fig. 7b).

#### Effect of TI phytochemical metabolites on CaCo-2 cell migration

**Figure 8.**
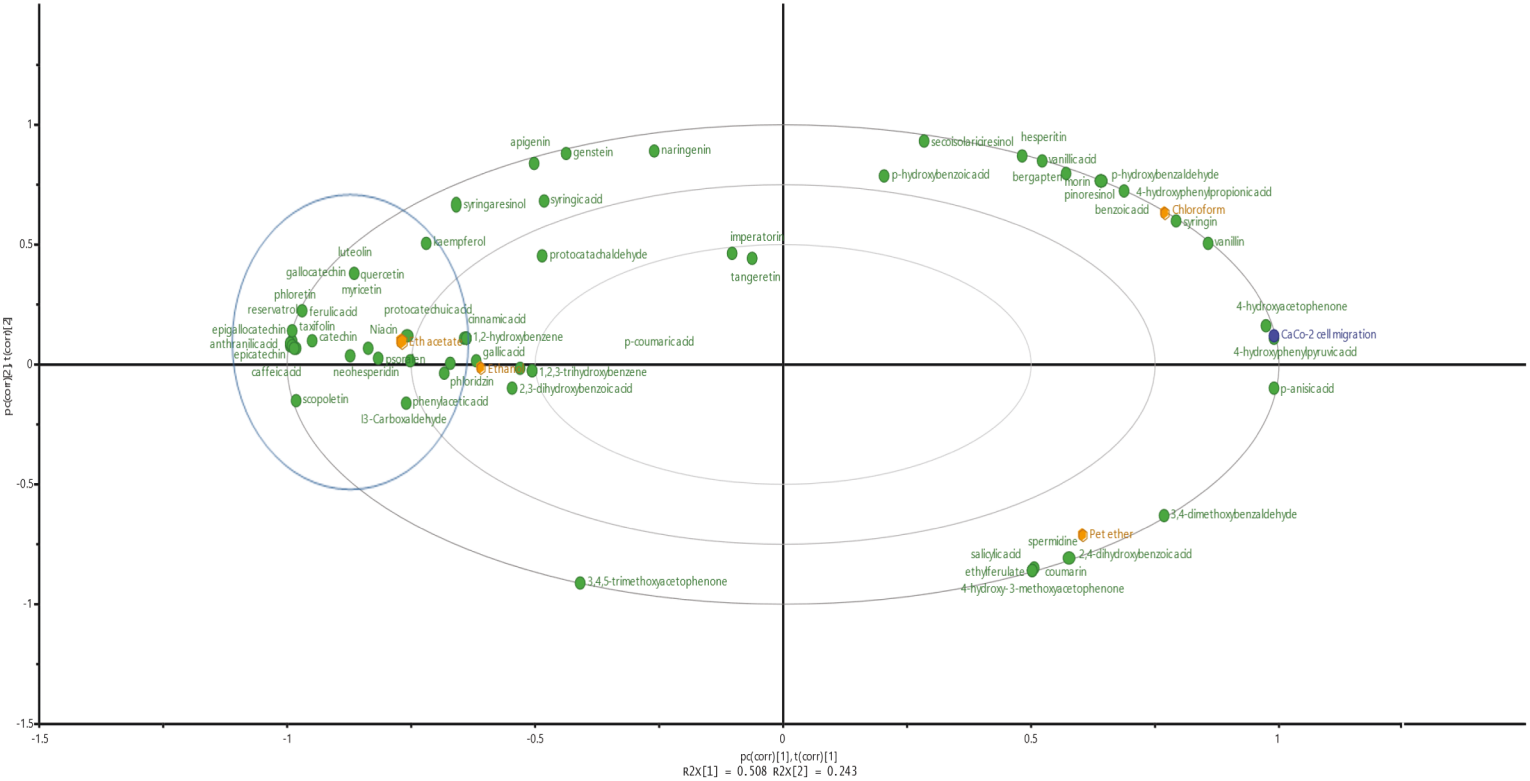
Association between TI extracts and CaCo-2 cell migration after 24-h treatments with TI extracts. The plot was colour coded as: deep blue for cell migration; green for phytochemical compounds; and orange for TI extracts.

TIEE or TIEaE significantly (*P* < .001) inhibited migration in CaCo-2 cells after 2 or 7 days, while cell migration was unaffected by TIPE and TICE (Fig. 7a). When the impact of specific phytochemicals on cell migration was analysed by PLS-DA, the inhibitory effect of TIEE or TIEaE was associated with the presence of anthranilic acid, caffeic acid, epicatechin, epigallocatechin, gallocatechin, kaempferol, luteolin, myricetin, neohesperidin, niacin, phenylacetic acid, phloretin, psolaren, and resveratrol Fig. 8 [7].

## Discussion

According to the American Cancer Society, cancer causes 2–3% of deaths annually throughout the world which accounts for about 10 million deaths worldwide [3,26]. Due to the global socioeconomic impact of cancer, numerous strategies have been used in cancer therapy and management which include chemotherapy, radiotherapy, and surgery [27,28]. Due to the many side effects associated with conventional drug treatment, natural products have been explored as treatment alternatives. For instance, plant alkaloids such as paclitaxel and vinblastine have been used for the treatment of various malignancies including brain, breast, cervical, lung, and pancreatic cancers [29]. However, these treatment methods are usually linked with severe side effects with cancerous cells gradually developing resistance against treatment [27]. Hence, cancer researchers continue to seek new and safer approaches to improve the treatment of cancer [30].

Currently, there are no data on the effect of TI on colon cancer. In African traditional medicine, TI is used for the treatment of diuresis, malaria, and ulcers [8,9] and as an anti-inflammatory, anti-nociceptive, nephro-, and hepatoprotective agent in animal models [13–15]. Therefore, in this novel study, we investigated the use of TI as a potential anticancer agent in human colon cells *in vitro* and to determine whether TI acts differently against a model of cancer (CaCo-2) and normal colon (NCM460) cells.

Different solvent extracts of TI have been shown to yield different phytochemical profiles [7]. Hence, this study established the activities of four TI extracts: chloroform extract (TICE), ethanol extract (TIEE), ethyl acetate extract (TIEaE), or petroleum ether extract (TIPE) on several primary biomarkers of cancer: antioxidant activity, cell death, cell growth, DNA damage, DNA repair, and cell migration.

Antioxidants have been reported to protect against damaged induced by free radicals and inhibit the carcinogenesis process by preventing initiation, development, and progress of the process. They show anticancer properties through the induction of apoptosis, inhibition of continuous cellular proliferation, angiogenesis, and metastasis [6,24,31].

DPPH is a widely used assay for assessing the antioxidant status of new drugs [32]. Due to the importance of metal ions in catalysing biological processes, the ferric-reducing antioxidant power (FRAP) assay was also used to evaluate the antioxidant activity of TI extracts. TIEE and TIEaE had significantly (*P* = 0.001) higher antioxidant effects on scavenging DPPH radicals and reducing ferric ions when compared with TICE and TIPE. The higher antioxidant activity shown by TIEE and TIEaE is likely due to the presence of higher amounts of flavonoids and phenolic phytochemical compounds such as catechin, epicatechin, gallic acid, quercetin, and resveratrol than in TICE and TIPE [7]. In similar studies, flavonoids and polyphenols such as catechin, epicatechin, quercetin, and resveratrol have been shown to possess high antioxidant activity [33], while benzoic acids such as gallic acid also possess a high DPPH scavenging activity [34].

In this study, increasing concentrations of TI extract (0–5 mg/ml) were used to evaluate the impact of TI on human colon cell survival characteristics. All four TI extracts showed a concentration-dependent negative effect on viability and growth and were toxic at concentrations ≥ 1 mg/ml. Most importantly, the colon cancer cell model showed more susceptibility towards the TI extracts than the normal colon cells, observed as an approximately two times increase in the death of CaCo-2 cells (IC_50_ values) when compared with the NCM460 cells. No such obvious difference in the differential effect of TI extracts on colonocyte cell growth was observed.

To the best of our knowledge, this is the first study investigating the potential anticancer properties of TI *in vitro* and comparing directly the effects on transformed versus non-malignantly transformed cells. Hence, previous studies using natural products and natural phytochemicals are used to discuss these findings. Ponou *et al*. (2010) investigated the cytotoxic effects of bioactive compounds from TI by administering 0–200 µM of arjungenin, arjunic acid, betulinic acid, ivorenosides (A, B or C), oleanolic acid or sericoside to human breast (MDA-MB-231), prostate (PC3), colon (HCT116), and brain (T98G) cancer cells for 24 h. Singly administered ivorenosides (A, B, or C) and sericoside at 200 µM reduced proliferation across all cell lines by 50–80%. Ivorenoside A was found to be the most toxic metabolite against MDA-MB-231 and HCT116 cell lines, with IC_50_ values of 3.96 and 3.43 µM, respectively [35]. This study did not measure these specific metabolites. TI extracts have been quantitatively profiled and shown to contain phytochemicals including myricetin and quercetin which might have contributed to the dose-dependent decrease in the colon cells viability, but the metabolites were not tested individually to confirm this prediction [7]. Conversely, administering increasing concentrations (1–10 µM) of falcarinol (a polyacetylene isolated from carrots) to CaCo-2 cells for 72 h increased cell proliferation by approximately 30–80% [31].

DNA damage and genomic instability are fundamental to the development of cancer and ultimately direct the cells to divide and grow continuously [3]. Here, the four TI extracts were used to determine their influence on genomic stability as DNA SSB and SBR in normal colon cells versus their cancerous counterparts.

Similar to the effects on cell viability and growth, all four TI extracts showed a concentration-dependent inverse association on colon cell genomic stability. Overall, TI extracts were more DNA damage-inducing in the colon cancer cell model compared with the non-malignant colon cells, with an approximate three times increase in DNA SSB in Caco-2 cells compared with the normal colon cells.

In similar studies investigating the genotoxicity of natural products, treatment of CaCo-2 cells with falcarinol at concentrations > 10 µM for 72 h significantly (*P* < .001) increased DNA SSB by > 90% [31]. CaCo-2 cells exposed to increasing concentrations (0–2500 µM) of flavonoids (including myricetin, quercetin, and silymarin) for 18 h had a significant induction (*P* < .01) in DNA SSB (approximately 20, 40, and 100% for silymarin, quercetin, and myricetin, respectively) as compared to the controls [36]. These individual compounds (except silymarin) have been identified in the TI extracts [7]. However, there are no data showing the impact of whole TI extracts on genomic stability in colon cells. The ability of TIEE, TICE, or TIPE to induce DNA damage specifically in cancer cells is an advantage for the potential search for anticancer drugs. A study by Collin *et al*. (1995) found that different cell types show varying responses to DNA damage caused by H_2_O_2_. This is attributed to differences in cellular Fe^2+^ or NAD(P)H levels, which impact the functions of various cellular antioxidant enzymes including catalase and glutathione peroxidase and influence the ability of the cells to resist oxidative stress [37].

Normal cells have checkpoints in the cell proliferation cycle that safeguard genomic integrity [36]. These checkpoint mechanisms help to regulate genetic material through a series of activities which may include transduction of information, sensing of DNA damage and ultimately triggering effector responses to control DNA damage [38]. When conditions are unfavourable for growth or there is irreparable DNA damage in the cells, apoptosis is triggered to remove the damaged cells [36]. In cancer cells, however, these cellular control mechanisms are inhibited, resulting in the accumulation of damaged genetic material and consequently, continuous division and growth of abnormal cells [38]. Most therapeutic agents used in the treatment of cancer eliminate cancerous cells by directly or indirectly inducing checkpoint-mediated controls, causing non-selective DNA damage which accounts for their toxicity in both cancerous and normal cells [36]. Such limited selective toxicity to cancer cells and non-specific damage to DNA largely accounts for the severe adverse effects mostly seen in cancer therapy [36].

Although direct damage to DNA is an essential element of cytotoxicity, the relative toxicity of potential anti-tumour drugs suggests that the magnitude of DNA damage is not an indicator of overall cellular toxicity [39] and that the extent of DNA repair in cells after damage will affect overall genotoxicity [40]. Therefore, this study determined the influence of TI extracts on DNA repair as a hallmark of cancer in the colon cells.

A non-cyto- and non-genotoxic concentration of TI (0.25 mg/ml) was used to measure the DNA SBR activity in the colon cells after inducing DNA SSB with H_2_O_2_. As expected, there was a significant increase in DNA SSB in DMSO control cells after exposure to H_2_O_2_ which was followed by a time-dependent decrease in DNA SSBs after 30 and 60 min in both colon cell lines. NCM460 cells showed a higher endogenous or background DNA SBR as compared to CaCo-2 cells. Pretreatment with TIEaE induced DNA repair in NCM460 after 60 min when compared with CaCo-2 cells (*P* = .001). The extent of removal of strand breaks is less, indicating a lower repair rate overall in the TI extract pre-treated group.

PLS-DA was used to assess the impact of the phytochemicals present in the TIEaE associated with the inhibitory effects. Inhibition of DNA SBR in CaCo-2 cells was significantly associated with 4-hydroxy-3-methoxyphenylpropionic acid, hydroxytyrosol, and indole-3-acetic acid, present exclusively in TIEaE but not in TICE, TIEE, or TIPE [7].

There are no available data on the effect of TI extracts on DNA SBR activity. However, in related studies, De Melo *et al*. (2004) treated neutrophils with 1 mM indole-3-acetic acid for 12 h and observed cell death which resulted from the induction of loss in cell membrane integrity and DNA fragmentation [41]. Folkes and Wardman (2001) proposed that the effect of indole-3-acetic acid to cause cytotoxicity may be due to the ability to form 3-methylene-2-oxindole, which conjugates with protein thiols and DNA bases [42]. Thus, indole-3-acetic acid, which is present in TIEaE, could serve as a potential anti-tumour agent as it is well tolerated in normal cells but toxic to tumour cells [42,43].

The effect of *Aronia melanocarpa*, *Chaenomeles superba*, and *Cornus mas* extracts on DNA SBR in CaCo-2 cells was investigated by treating the cells with extract concentrations of 0–10% (v/v) for 1 h. DNA damage was induced in a dose-dependent manner while non-toxic concentrations (0.04–0.08%) significantly (*P* < .05) induced DNA SBR by approximately 40% after 60 min of exposure to H_2_O_2_ [44]. Similarly, treatment of CaCo-2 cells with 50 µg/ml of *Viburnum opulus* fruit extract for 60–120 min showed > 90% induction in DNA repair after 120 min of exposure to H_2_O_2_ [45]. The induction of DNA SBR by polyphenols is via promoting the activity of DNA repair enzymes [46]. According to Hengel *et al*. (2017), healthy cells employ the process of DNA repair to preserve their genetic integrity and mitigate the effects of ageing, and lower the risk of developing cancer [47].

Normal cells use the mechanism of DNA repair to remove damaged DNA and maintain their genomic integrity and reduce the risk of cancer [47]. However, cancer cells lose this characteristic of normal cells to remove DNA damage from its genomic material [47]. The ability of TI extract to selectively inhibit DNA repair and cause lethality in colon cancer cells but not in normal colon cells as observed here, may be a key feature as a promising anticancer agent.

Cell invasiveness/migration is the single most important feature that differentiates malignant and benign cells [48]. Contrary to benign lesions, malignant cells develop mechanisms that enable them to migrate from the original point of development and form new colonies in different sites of an organism [49]. However, after an injury to epithelium, cells proliferate, differentiate, and migrate to promote wound healing as shown previously in a study with CaCo-2 and NCM460 cells [50].

As the focus of this study is examining the effect of different TI extracts on colon cancer. The malignantly transformed human colon cancer cells (CaCo-2 cells only) were used as a model for radial migration to determine the impact of TI extracts on cell migration. Besides, the study aimed to measure cell migration rather than cell proliferation, hydroxyurea (HU) was used as a growth inhibitor to negate the influence of growth on the migration assay. In control cells (DMSO or HU), a time-dependent increase in migration for up to 7 days was observed as shown by the leading cellular edges of migration. Treatment with TI extracts showed differential effects on cell migration. Cells exposed to TIEaE or TIEE displayed a greater than 70% inhibition in migration at 2–7 days as compared to the controls or other extracts.

This is the first study to comprehensively show the ability of complex (crude) extract metabolites (rather than single metabolite) on cell migration. Those metabolites present in TIEaE or TIEE extracts, calculated to have the greatest impact on migration include benzenes (especially 1,2-dihydroxybenzene), benzoic acids (especially anthranilic acid), cinnamic acids (especially caffeic acid and cinnamic acid), and flavonoids (especially epicatechin, epigallocatechin, gallocatechin, kaempferol, luteolin, myricetin, neohesperidin, niacin, phenylacetic acid, phloretin, psolaren, and resveratrol) [7].

In agreement with these findings, treatment of radioresistant lung cancer (A549 or A549-IR) cells with 100 µM of myricetin for up to 2 days significantly (*P* < .001) reduced migration [51]. Taxifolin (30 µm) significantly (*P* < .001) decreased the migratory ability of human osteosarcoma cell lines (U2OS and Saos-2) after 2 days of treatment [52]. Human malignant glioma (LN-18 and T98G) cells treated with 300 µM of benzoic acid or N- [3,4-dimethoxycinnamoyl]-anthranilic acid for 4 days showed inhibited migration by approximately 60% in both cell lines as compared to the respective controls [53]. Treating human colon (Lovo) cells with 5 µM of cinnamic acid for 1–2 days significantly (*P* < .05) reduced the migration of the cells by approximately 78% as compared to the control [54].

This first *in vitro* ‘proof of concept’ study demonstrates mechanistically the ability of TI ethanol and ethyl acetate extracts to induce higher cyto- and genotoxicity in human colon cancer cells when compared with normal cells, and to strongly inhibit the migration of cancer cells, indicating the potential of TI as an anti-cancer treatment. However, further work, using *in vitro* and *in vivo* models, is required to establish the physiological-relevant and effective concentrations of TI, the effect of TI on endogenous xenobiotic metabolism, and any long-term toxicities.

## Conclusions

TI extracts from different solvents showed differential antioxidant activity and specific anticancer effects. TI extracts were equally cyto- and genotoxic to both normal and cancerous colon cells at higher concentrations. However, cancerous colon cells were more susceptible to the TI extracts at lower concentrations. The anticancer potential observed by the TI extracts, as evidenced by induction of DNA damage, inhibition of DNA repair, and inhibition of cell migration may be due to the presence of certain benzenes, benzoic acids, cinnamic acids, and flavonoids.

## Data Availability

The data can be made available from the corresponding author on reasonable request.
